# Clinicopathological features and prognosis of mucinous breast carcinoma with a micropapillary structure

**DOI:** 10.1111/1759-7714.15480

**Published:** 2024-11-09

**Authors:** Beibei Yang, Menglu Shen, Bo Sun, Jing Zhao, Meng Wang

**Affiliations:** ^1^ Department of Breast Cancer, Tianjin Medical University Cancer Institute & Hospital, National Clinical Research Center for Cancer/Tianjin's Clinical Research Center for Cancer/Key Laboratory of Breast Cancer Prevention and Therapy Tianjin Medical University, Ministry of Education Tianjin China; ^2^ Department of Lung Cancer, Tianjin Medical University Cancer Institute and Hospital National Clinical Research Center for Cancer, Key Laboratory of Cancer Prevention and Therapy, Tianjin’s Clinical Research Center for Cancer, Tianjin Lung Cancer Center Tianjin China

**Keywords:** breast cancer, clinicopathological features, lymph node metastasis, mucinous carcinoma, prognosis

## Abstract

**Objective:**

To conduct a comparative analysis of clinicopathological data between mucinous micropapillary breast carcinoma (MUMPC) and pure mucinous carcinoma (PMC) without a micropapillary structure to elucidate the distinctive clinicopathological characteristics of MUMPC and their impact on prognosis.

**Methods:**

This retrospective analysis included 325 patients diagnosed with mammary mucinous carcinoma admitted to Tianjin Cancer Hospital between July 2014 and December 2019, including 197 patients with MUMPC and 128 patients with PMC without a micropapillary structure. Clinicopathological features were compared, and factors influencing the prognosis of MUMPC were analyzed. Survival analysis was conducted using the Kaplan–Meier method, and univariate and multivariate prognostic analyses for MUMPC were performed using the Cox proportional hazard regression model.

**Results:**

The median follow‐up period was 76 months. In the MUMPC and PMC groups, the disease‐free survival (DFS) rates at 3, 5, and 7 years were 95.4%, 90.4%, 89.8%, and 100%, 98.4%, and 96.9%, respectively, with a statistically significant difference between the two groups (*p* = 0.009). Tumor, node, and metastasis (TNM) stage, lymph node metastasis, and endocrine treatment were significant factors influencing the prognosis of the MUMPC group (*p* < 0.001). Multivariate analysis revealed that lymph node metastasis and endocrine therapy were independent prognostic factors in patients with MUMPC (*p* < 0.001). Compared with PMC, the MUMPC group exhibited a higher prevalence of HER2 (11.2% vs. 3.1%, *p* = 0.009) and Ki‐67 overexpression (79.7% vs. 60.2%, *p* < 0.001).

**Conclusion:**

The lymph node stage is the most crucial clinicopathological feature of MUMPC. Endocrine treatment strategy is an independent risk factor affecting the prognosis of MUMPC.

## Graphical abstract

The MUMPC and PMC groups exhibited significant differences in the rate of DFS at 3, 5, and 7 years (*p* = 0.009). The lymph node stage is the most crucial clinicopathological feature of MUMPC. Endocrine treatment strategy is an independent risk factor affecting the prognosis of MUMPC. Compared with PMC, the MUMPC group exhibited a higher prevalence of HER2 (*p* = 0.009) and Ki‐67 overexpression (*p* < 0.001).

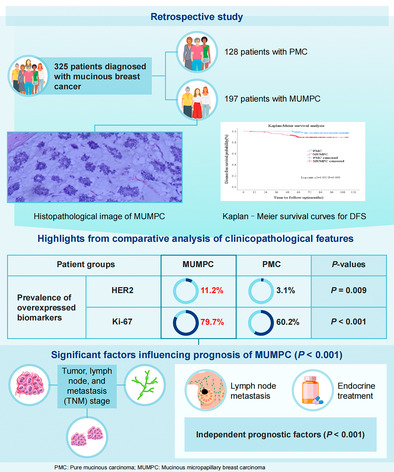

## INTRODUCTION

**FIGURE 1:**
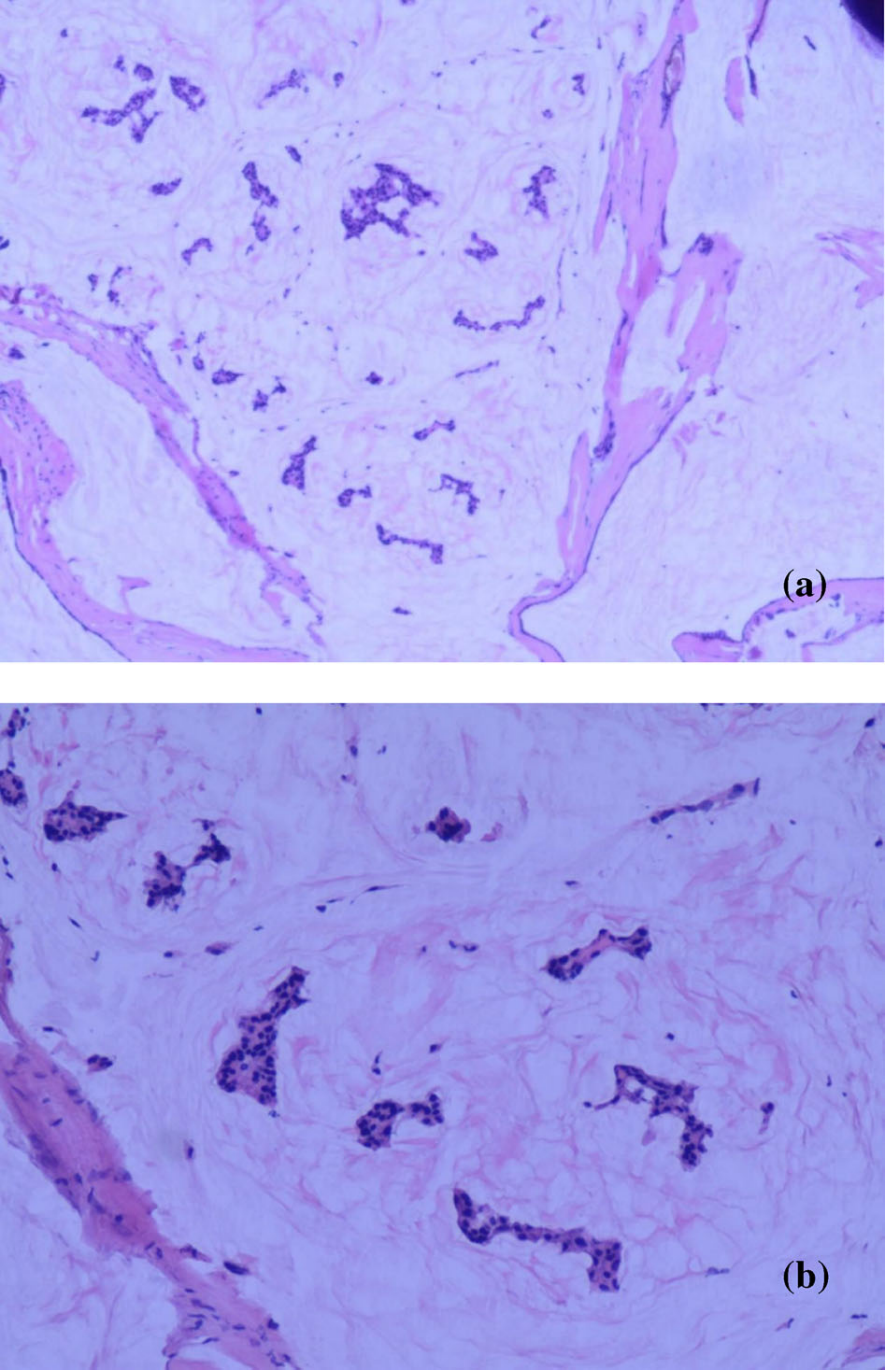
Histopathological image of MBC. Tumor cells “nests” floating in abundant extracellular mucus (a:H&E × 40; b:H&E:×100).

Mucinous micropapillary breast carcinoma (MUMPC), initially reported by Ng^1^ in 2002, is characterized by the presence of tumor cells arranged in a micropapillary architecture within mucinous cancer. This histopathological variant represents a subtype of mucinous breast carcinoma (MBC).^2^, ^3^, ^4^ MBC is generally considered an indolent tumor with a favorable prognosis, accounting for 1%–4% of all breast cancers.^5^, ^6^, ^7^, ^8^ Pure mucinous breast carcinoma (PMC) is a subtype of MBC in which >90% of the tumor volume consists of mucinous components, as shown in Figure 1. Conversely, invasive micropapillary carcinoma (IMPC) is a distinct subtype of breast cancer. Morphologically, micropapillae are composed of solid clusters or ring‐shaped arrangements of tumor cells separated by empty spaces and display an “inside‐out pattern.” IMPC exhibits histopathological features characterized by a micropapillary structure and a significant propensity for lymphatic vascular invasion and lymph node metastasis, leading to an unfavorable prognosis.^9^, ^10^, ^11^ Compared with nonspecific invasive breast cancers, PMC types demonstrate better prognoses, whereas IMPC is associated with poorer outcomes. In terms of histological morphology, the arrangement of MUMPC is similar to that of IMPC. The major difference between MUMPC and IMPC is that MUMPC tumor cells float in a large amount of mucus.^12^ MUMPC has been recognized by the WHO as a novel subtype of MBC. However, some scholars argue that MUMPC should be classified as a subtype of IMPC.

MUMPC has dual morphological structures that resemble both PMC and IMPC. However, due to its low incidence, its clinicopathological features remain unclear.

This study aimed to collect clinicopathological data from patients with MUMPC and compare them with those from patients with PMC to explore further the clinicopathological characteristics of MUMPC and their impact on patient prognosis. These findings aimed to enhance our understanding of this particular tumor type, with the goal of proposing optimized diagnostic approaches, treatment guidelines, and prognostic evaluation criteria for clinical practice.

## METHODS

### Participants

A retrospective analysis was conducted on a cohort of 325 patients diagnosed with MBC who were admitted to the Tianjin Cancer Hospital between July 2014 and December 2019. Initial treatment excluded patients with remote metastatic breast cancer. Among them, 197 patients with MUMPC were enrolled in the study group, whereas 128 patients with PMC without a micropapillary structure were enrolled in the control group. The age range of the participants was 22–88 years, with a median age of 52 years. All patients underwent standard breast cancer surgery, including breast‐conserving surgery (BCS) and sentinel lymph node biopsy (SLNB). Demographic factors, including age, family history, menstrual status, and tumor size, were meticulously documented. Additionally, crucial clinical parameters such as lymph node metastasis, estrogen receptor (ER) and progesterone receptor (PR) expression levels, human epidermal growth factor receptor‐2 (HER2) status, antigen Ki‐67 associated with tumor proliferating cells, p53 tumor suppressor expression level, surgical method, and administration of chemotherapy, radiotherapy, endocrine, or targeted therapy regimens were comprehensively recorded. The eighth edition of the American Joint Committee on Cancer Breast Cancer staging system was used to determine the clinical TNM stage.

The present study was approved by the Medical Ethics Committee of Tianjin Cancer Hospital, and informed consent was obtained from all participating patients.

### Methodology

**FIGURE 2:**
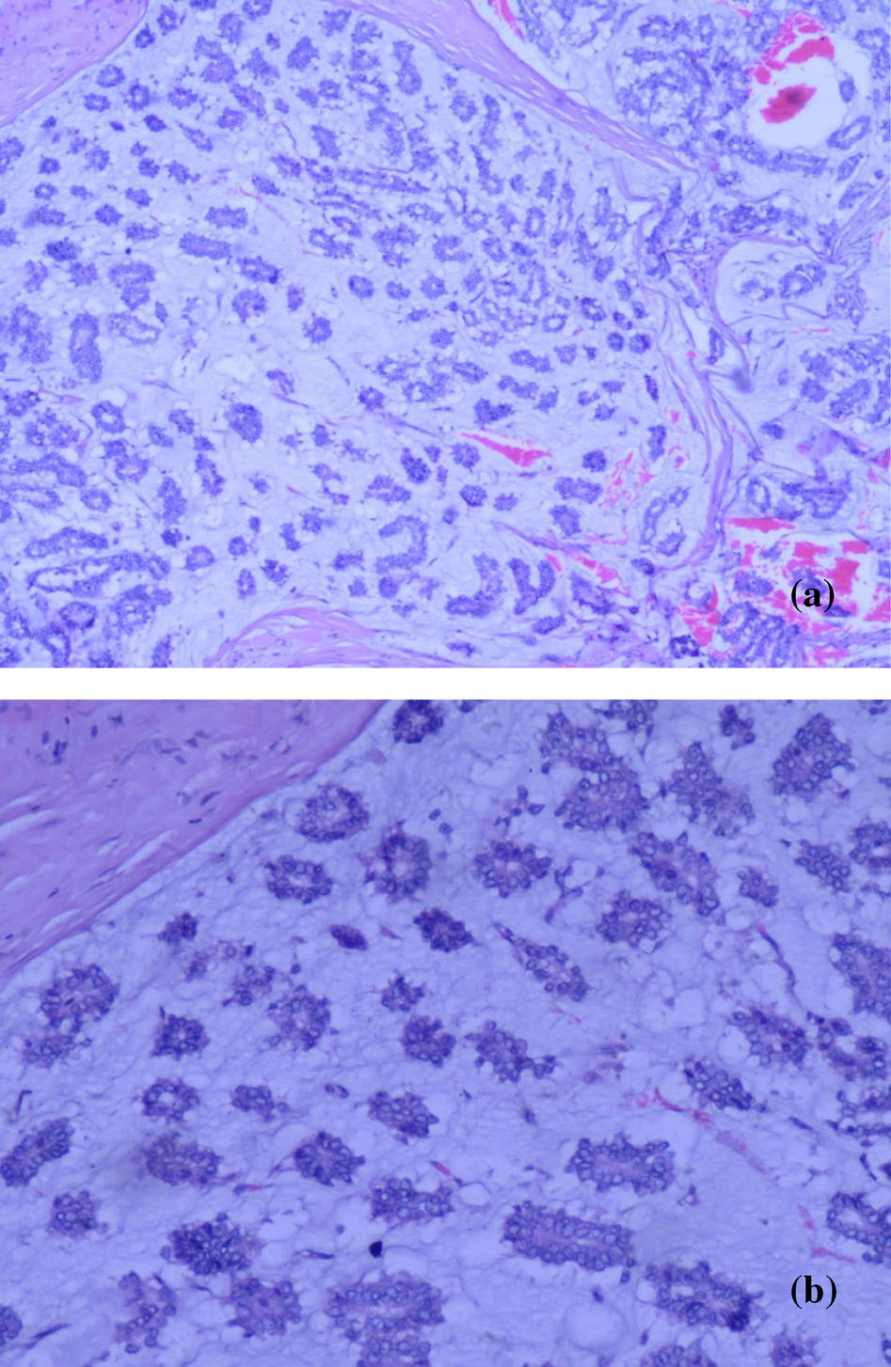
Histopathological image of MUMPC. Tumor cells were arranged in a micropapillary, pseudoglandular tubular shape within mucin‐filled stromal compartments (a:H&E × 40; b:H&E:×100).

The diagnostic micropapillary structure of MUMPC refers to the IMPC diagnostic criteria of the World Health Organization (WHO) Classification of Breast Tumors (2012), where tumor cells are arranged in micropapillary or pseudoglandular tubes, or both, as shown in Figure 2: Epithelial membrane antigen (EMA) peripheral staining pattern of “polarity reversal.” Morphologically, MUMPC resembles PMC. However, limited research has been conducted to determine the optimal cutoff value for the percentage of micropapillae (MP%) in mucinous carcinoma when diagnosing MUMPC. Shet et al.^13^ and Xu et al.^14^ defined MUMPC as a mucinous carcinoma with diffuse micropapillary features, or a MP% of >90%. A retrospective cohort study conducted by Liu et al. demonstrated that MUMPC with micropapillary composition >50% exhibited a significantly worse prognosis.^12^ The MP% of MUMPC included in Sun et al.'s study ranged from 5% to 100%, with 75% of the samples having a MP% of ≥50%.^2^ In the current study, the MP% range for diagnosing MUMPC was set to >50%.

MUMPC is defined as a mammary mucous carcinoma with a micropapillary component comprising >50% of tumor epithelial cells.

The immunohistochemical staining and interpretation of ER, PR, and HER2 were performed according to the American Society of Clinical Oncology/College of American Pathologists guidelines. Tumors were considered positive for ER or PR if strong immunoreactivity was observed in more than 1% of tumor nuclei.^15^ A PR value of <20% indicated low PR expression. For HER2, strong and intact cell membrane staining (3+) in >10% of infiltrating cancer cells was defined as positive, while 0 and 1+ staining were considered negative. Weak to moderate strength, and complete cell membrane staining (>10% of infiltrated cells), defined as 2+, can be detected through fluorescence in situ hybridization (FISH). HER2‐positive and negative groups were categorized based on the presence or absence of HER2 gene amplification.^16^ ER‐positive, PR ≥20%, HER2 negative and Ki‐67 <14% were classified as luminal A; nonluminal A with positive ER/PR was defined as luminal B; ER, PR, and HER‐negative were assessed as triple‐negative; positive HER2 was classified as HER2 overexpression. The Ki‐67 and p53 staining percentage in the tumor region was evaluated and documented as a continuous variable. A Ki‐67 staining percentage of <14% indicated low Ki‐67 expression; p53 status was classified as negative using a cutoff of 1%.

Follow‐up assessments were conducted in outpatient clinics, via telephone calls, and in inpatient clinics. The follow‐up period commenced on the day of breast cancer surgery and was concluded upon the occurrence of breast cancer‐related death, local recurrence, distant metastasis, or completion of follow‐up until March 2024. The follow‐up duration ranged from 7 months to 114 months, with a median follow‐up time of 76 months. Comprehensive aspects such as recurrence, metastasis, survival status, and ongoing treatment were included in the follow‐up evaluations. Given the favorable prognosis associated with MBC, disease‐free survival (DFS) served as the primary outcome measure for this study.

### Statistical analysis

Statistical analysis was conducted using SPSS 25.0, employing the appropriate statistical methods. Data are expressed as percentages (%). Clinicopathological features of MUMPC and PMC were compared using the Pearson's chi‐square and Fisher's exact tests. Survival analysis was performed using the Kaplan–Meier method. The univariate and multivariate prognoses of MUMPC were analyzed using the Cox proportional hazards regression model. Statistical significance was defined as *p* < 0.05.

## RESULTS

### Comparison of clinicopathological features between groups

All 325 patients were followed up for 7–114 months, with a median follow‐up period of 76 months. There was no significant difference in the age of onset between the PMC and MUMPC groups (*p* = 0.594). However, a higher proportion of premenopausal patients was observed in the MUMPC group (*p* = 0.049). There was no significant difference in tumor size between the two groups (median: 2.54 vs. 2.26 cm; *p* = 0.129). Nevertheless, the MUMPC group exhibited a higher incidence of lymph node metastasis (33% vs. 3.9%; *p* < 0.001) and advanced TNM stage (*p* < 0.001). Regarding molecular typing, the proportion of ER positivity was comparable between the two groups, with over 97% of the patients in both groups exhibiting ER positivity. However, among PR‐positive patients, MUMPC demonstrated a higher prevalence of low PR expression (*p* = 0.003). Furthermore, compared with the PMC group, the MUMPC group exhibited a greater incidence of HER2 overexpression (11.2% vs. 3.1%, *p* = 0.009) and a higher level of Ki‐67 expression (79.7% vs. 60.2%, *p* < 0.001).

Regarding treatment, >50% of patients in the PMC group did not receive chemotherapy, which was significantly higher than that in the MUMPC group (52.3% vs. 23.9%, *p* < 0.001). Among the patients who underwent chemotherapy, the proportion receiving neoadjuvant chemotherapy was higher in the MUMPC group than in the PMC group (9.1% vs. 2.3%, *p* = 0.015). Although there was no statistically significant difference in the rate of breast conservation (16.2% vs. 13.3%, *p* = 0.466), the MUMPC group had a significantly higher proportion of patients who received radiotherapy (33.0% vs. 12.5%, *p* < 0.001). Due to the higher prevalence of HER2 overexpression in the MUMPC group, there was increased use of anti‐HER2 therapy among the patients (10.7% vs. 2.3%, *p* = 0.005). Among those undergoing endocrine therapy, the MUMPC group had a lower tamoxifen ratio for single‐drug administration than the PMC group, while there was an increase in the proportion of patients receiving step‐up therapy, such as ovarian function inhibitors, aromatase inhibitors, or chemotherapy (*p* = 0.018), as presented in Table 1.

**TABLE 1:** Comparison of the clinicopathological features between the two groups.

Variables	MUMPC (*N* = 197)		MBC (*N* = 128)		Chi‐square	*p* value
	No	Percent (%)	No	Percent (%)		
Age					0.284	0.594
<40	32	16.2	18	14.1		
≥40	165	83.8	110	85.9		
Family history					2.475	0.116
No	185	94.0	125	97.7		
Yes	12	6.0	3	2.3		
Menopause					3.870	0.049*
No	102	51.8	52	40.6		
Yes	95	48.2	76	59.4		
T stage					5.024	0.158
T1	77	39.1	65	50.8		
T2	110	55.8	56	43.8		
T3	8	4.1	5	3.9		
T4	2	1.0	2	1.5		
N stage					44.547	<0.001**
N0	131	67.0	123	96.1		
N1	49	25.4	5	3.9		
N2	9	3.6	0			
N3	8	4.0	0			
pTNM					17.464	<0.001**
I	61	31.5	65	50.8		
II	114	57.9	60	46.9		
III	22	10.6	3	2.3		
ER					0.349	0.555
Negative	5	2.5	2	1.6		
Positive	192	97.5	126	98.4		
PR					11.827	0.003**
Negative	17	8.6	8	6.3		
Low expression	52	26.4	15	11.7		
High expression	128	65.0	105	82.0		
HER2					6.819	0.009**
Negative	175	88.8	124	96.9		
Positive	22	11.2	4	3.1		
Ki‐67					14.693	<0.001**
Low expression	40	20.3	51	39.8		
High expression	157	79.7	77	60.2		
P53					2.494	0.114
Negative	85	43.1	44	34.4		
Positive	112	56.9	84	65.6		
Molecular subtypes					27.170	<0.001**
Luminal A	31	15.7	50	39.1		
Luminal B	143	72.6	72	56.3		
HER2	22	11.2	4	3.1		
TN	1	0.5	2	1.5		
Breast surgery					0.532	0.466
Mastectomy	165	83.8	111	86.7		
Breast‐conserving	32	16.2	17	13.3		
Lymph node operation					0.062	0.803
SLNB	59	29.9	40	31.3		
Dissection	138	70.1	88	68.7		
Chemotherapy					27.646	<0.001**
No	47	23.9	67	52.3		
Yes	150	76.1	61	47.7		
Radiotherapy					17.417	<0.001**
No	132	67.0	112	87.5		
Yes	65	33.0	16	12.5		
Targeted therapy					7.846	0.005**
No	176	89.3	125	97.7		
Yes	21	10.7	3	2.3		
Endocrine therapy					10.243	<0.001**
No	8	4.1	8	6.3		
Tamoxifen	50	25.4	50	39.1		
AI	116	58.9	63	49.2		
OFS + Tamoxifen	3	1.5	0	0		
OFS + AI	20	10.1	7	5.4		
Neoadjuvant therapy					5.924	0.015*
No	179	90.9	125	97.7		
Yes	18	9.1	3	2.3		
Recurrence					5.602	0.018*
No	177	89.8	124	96.9		
Yes	20	10.2	4	3.1		

Abbreviations: AI, aromatase inhibitors; ER, estrogen receptor; HER2, human epidermal growth factor receptor‐2; Ki‐67 Low expression, Ki‐67 < 14%; M, metastasis; MBC, mucinous breast carcinoma; MUMPC, mucinous micropapillary breast carcinoma; N, node; OFS, ovarian function suppression; p53 Negative, p53 < 1%; PR Low expression, 1% ≤ PR < 20%; PR, progesterone receptor; T, tumor.

* *p* < 0.05.

** *p* < 0.01.

### Risk factors for prognosis in MUMPC

Disease progression was observed in 20 patients in the MUMPC group, including ipsilateral chest wall involvement, ipsilateral axillary or supraclavicular lymph node metastasis, and distant organ metastases, including the bone (*n* = 6), lung (*n* = 3), liver (*n* = 1), and brain (*n* = 2). Univariate analysis of MUMPC prognosis revealed that age, family history, menstrual status, tumor size, molecular typing, chemoradiotherapy, and targeted therapy did not significantly affect the prognosis of patients with MUMPC (*p* > 0.05). Conversely, clinical TNM stage, lymph node metastasis, and endocrine therapy were identified as significant factors that influenced the prognosis of patients with MUMPC (*p* < 0.001) (Table 2). Furthermore, the results of multivariate analysis indicated that lymph node metastasis and endocrine treatment independently served as risk factors affecting the prognosis of patients with MUMPC (*p* < 0.001), as shown in Table 3.

**TABLE 2:** Univariate factor analysis of the disease‐free survival rate of MUMPC.

Variables	β	SE	Wald χ^2^	*p*	HR	HR 95% CI
Age	1.339	1.026	1.704	0.192	3.816	0.511–28.509
Family history	1.005	0.627	2.574	0.109	2.732	0.800–9.329
Menopause	−0.170	0.450	0.142	0.706	0.844	0.350–2.037
T stage	0.598	0.317	3.566	0.059	1.819	0.978–3.384
N stage	0.899	0.202	19.761	<0.001**	2.456	1.653‐3.651
pTNM	1.447	0.373	15.053	<0.001**	4.248	2.046‐8.822
ER	−0.610	1.026	0.353	0.552	0.543	0.073–4.060
PR	−0.306	0.316	0.939	0.333	0.736	0.396–1.368
HER2	0.357	0.626	0.325	0.569	1.429	0.419–4.879
Ki‐67	0.861	0.745	1.333	0.248	2.365	0.549–10.193
P53	0.342	0.469	0.533	0.465	1.408	0.562–3.530
Molecular subtypes	0.300	0.403	0.554	0.457	1.350	0.613–2.975
Breast surgery	−0.569	0.745	0.582	0.446	0.566	0.131–2.441
Lymph node operation	0.232	0.517	0.202	0.653	1.262	0.458–3.473
Chemotherapy	0.591	0.626	0.890	0.346	1.805	0.529–6.161
Radiotherapy	0.150	0.469	0.103	0.749	1.162	0.463–2.914
Targeted therapy	0.419	0.626	0.447	0.504	1.520	0.445–5.190
Endocrine therapy	−0.571	0.282	4.107	0.043*	0.565	0.325‐0.981
Neoadjuvant therapy	0.674	0.626	1.158	0.282	1.962	0.575–6.698

Abbreviations: AI, aromatase inhibitors; CI, confidence interval; ER, estrogen receptor; HER2, human epidermal growth factor receptor‐2; HR, hazard ratio; M, metastasis; MUMPC, mucinous micropapillary breast carcinoma; N, node; OFS, ovarian function suppression; PR, progesterone receptor; SE, standard error; T, tumor.

* *p* < 0.05.

** *p* < 0.01.

**TABLE 3:** Multivariate analysis of the disease‐free survival rate of MUMPC.

Variables	β	SE	Wald χ^2^	*p*	HR	HR 95% CI
N stage	0.789	0.370	4.557	0.033*	2.202	1.067–4.547
pTNM	0.493	0.559	0.778	0.378	1.637	0.547–4.900
Endocrine therapy	−0.708	0.298	5.634	0.018*	0.493	0.275–0.884

Abbreviations: CI, confidence interval; HR, hazard ratio; M, metastasis; MUMPC, mucinous micropapillary breast carcinoma; N, node; SE, standard error; T, tumor.

* *p* < 0.05.

### Survival analysis

**FIGURE 3:**
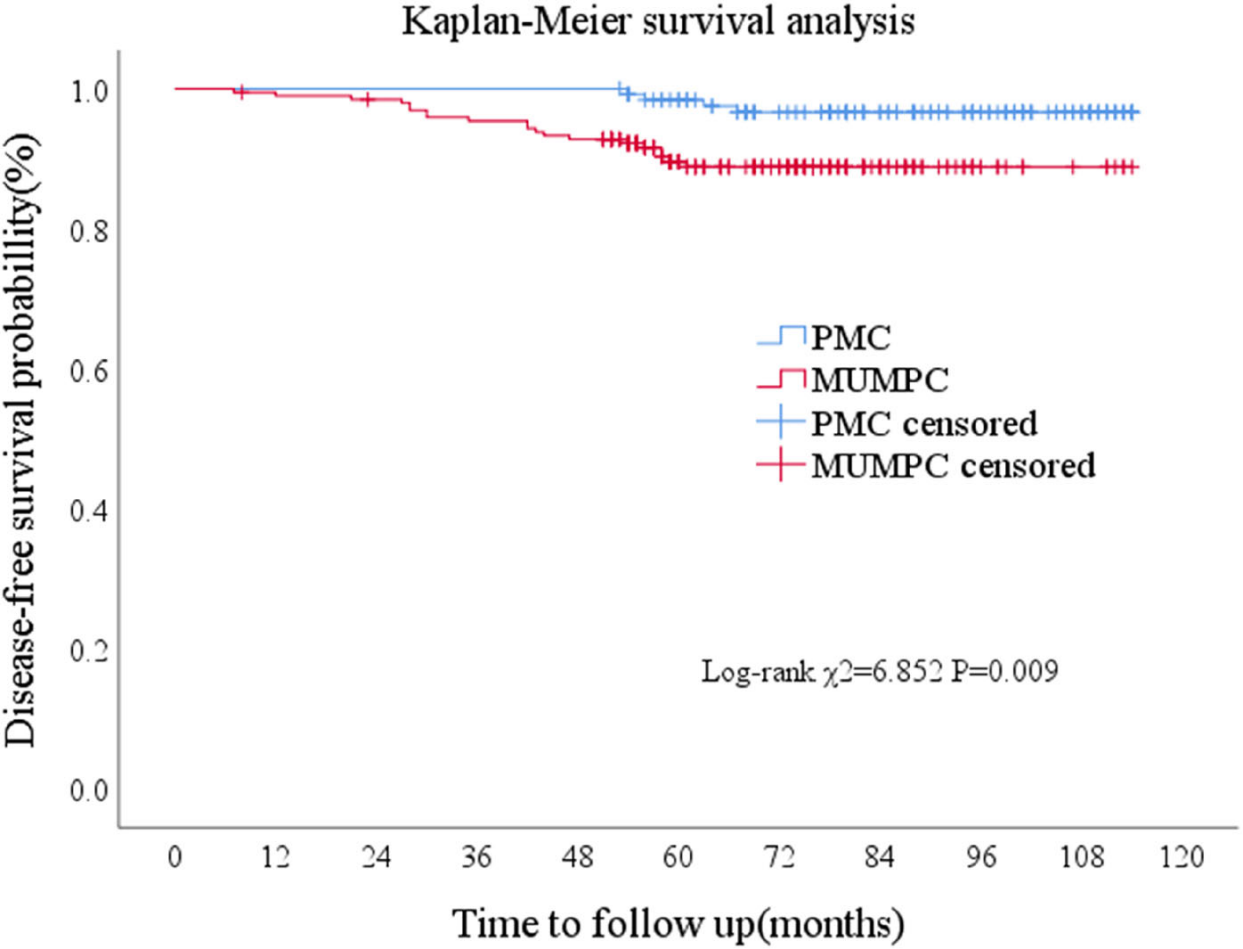
Kaplan–Meier survival curves for DFS. Disease‐free survival of patients with and without micropapillary structure. The graph shows DFS rates in MUMPC group were higher at the same time points in the PMC group, The difference between the two groups was statistically significant (*p* = 0.009).

DFS rates at 3, 5, and 7 years in the MUMPC group were 95.4%, 90.4%, and 89.8%, respectively. In contrast, DFS rates were higher at the same time points in the PMC group, with values of 100%, 98.4%, and 96.9%, respectively. The difference between the two groups was statistically significant (*p* = 0.009), as shown in Figure 3. The 3‐, 5‐, and 7‐year overall survival (OS) rates of patients with MUMPC were 100%, 98.5%, and 98.0%, respectively. The 3‐, 5‐, and 7‐year OS rates of patients with PMC were all 100%.

## DISCUSSION

In 2002, Ng^1^ first reported the micropapillary morphology of MUMPC, which WHO subsequently recognized as a novel MBC subtype. However, certain studies have proposed that MUMPC exhibits an arrangement similar to that of IMPC. Furthermore, the presence of micropapillary structures in MUMPC may be correlated with tumor invasiveness, including lymph node metastasis and lymphatic vessel invasion.

PMC primarily manifests in postmenopausal or older patients, with a prevalence rate of up to 70% among individuals aged ≥60 years and <0.4% among younger breast cancer cases.^17^, ^18^, ^19^ Several studies have indicated that young patients with breast cancer exhibit a higher degree of malignancy and a poorer prognosis.^20^, ^21^ Consequently, it is imperative to develop tailored treatment regimens for young women at a high risk of developing breast cancer. Compared with PMC, MUMPC generally exhibits a broader age range and predominantly affects younger women. However, in this study, no significant difference was observed in the age distribution between the MUMPC and PMC groups, with the median ages of onset being 52 and 51 years, respectively. This finding may be attributed to racial disparities. Furthermore, although there was no notable disparity in the proportion of young patients with breast cancer between the two groups, a higher proportion of premenopausal individuals was observed in the MUMPC group.

Regarding the comparison of tumor stages, patients with PMC exhibited low‐to‐moderate nuclear grade, rare lymphatic vessel invasion, and limited lymph node metastasis. This indolent behavior is associated with relatively low genomic instability, reduced proliferative activity, hormone receptor positivity, and minimal HER2 amplification.^22^, ^23^, ^24^ Furthermore, it has been proposed that the abundant presence of extracellular mucins acts as a physical barrier between tumor cells and the surrounding matrix, thereby impeding the diffusion of PMC.^25^, ^26^

The lymph node metastasis pathway is considered the most crucial route for breast cancer dissemination and is often associated with poor prognosis. MUMPC has a significantly higher incidence of axillary lymph node metastasis than PMC but a lower incidence than IMPC.^27^, ^28^, ^29^ In this study, the MUMPC group exhibited a higher rate of lymph node metastasis (33% vs. 3.9%, *p* < 0.001) and presented with a more advanced TNM stage (*p* < 0.001) than the PMC group. Univariate analysis demonstrated a significant association between clinical TNM stage and lymph node metastasis and the prognosis of patients with MUMPC (*p* < 0.001). Furthermore, multivariate analysis revealed that lymph node metastasis independently contributed to prognostic risk in patients with MUMPC (*p* < 0.001).

Di Saverio et al.^5^ showed that tumor size was an independent prognostic factor for PMC; however, it had limited predictive value, potentially due to the predominant presence of mucins occupying a significant portion of the tumor volume.^30^ Notably, no significant disparities in tumor size or breast‐conserving surgery rates were observed between the two groups. In the MUMPC group, two cases involved tumors infiltrating the chest wall and skin. However, neither recurrence nor metastasis occurred. These findings suggest that an increase in tumor size does not necessarily correlate with an elevated risk of recurrence and metastasis.

Most PMCs are classified as hormone receptor‐positive subtypes^31^; however, only a few studies have investigated the molecular typing of MUMPC. Some researchers suggest that the molecular typing of MUMPC is similar to that of PMC; however, compared with PMC, MUMPC tends to exhibit a higher histological grade and overexpression of HER2, p53, and Ki‐67, indicating a more aggressive phenotype.^14^, ^32^, ^33^, ^34^

The findings of this study are consistent with those of previous studies. The proportion of ER‐positive patients in both groups was similar, with >97% of the patients in each group exhibiting positive ER expression. However, the MUMPC group showed a higher percentage of low‐PR expression than the PMC group (*p* = 0.003). Additionally, the MUMPC group showed higher HER2 (11.2% vs. 3.1%, *p* = 0.009) and Ki‐67 (79.7% vs. 60.2%, *p* < 0.001) expression. International Ki‐67 in Breast Cancer Working Group (IKWG) confirms that Ki‐67 is clinically valuable as a prognostic marker, especially in patients with ER+ and HER2‐breast cancer.^35^ In clinical practice, patients with breast cancer exhibiting high Ki‐67 expression typically necessitate conventional chemotherapy, whereas those displaying low Ki‐67 expression may primarily benefit from surgical intervention and endocrine therapy. Consequently, the utilization of the Ki‐67 test enables clinicians to formulate a more individualized treatment plan for breast cancer patients.

In both groups, we observed significant differences between luminal A and B subtypes, with the former exhibiting a higher prevalence in PMC cases (15.7% vs. 39.1%, *p* < 0.001), whereas the latter was more common in patients with MUMPC (72.6% vs. 56.3%, *p* < 0.001). These findings have implications for treatment decisions, as evidenced by the higher proportion of adjuvant chemotherapy administration among patients with MUMPC (76.1%) than among patients with PMC (47.7%). This discrepancy may be attributed to the increased presence of the luminal B subtype of MUMPC, along with an elevated Ki‐67 proliferation index.

Regarding endocrine therapy, a retrospective analysis of 33 patients with MPCS revealed that the Oncotype DX 21 gene test exhibited a low recurrence score in 79% (26 of 33) of cases.^36^ The findings from this study imply that MUMPC might derive additional benefits from chemotherapy compared with PMC (*p* = 0.018). However, due to the limited sample size, there is currently insufficient evidence to support a relatively more aggressive treatment approach for patients with MUMPC, although there are clinical differences between MUMPC and PMC. The feasibility of genetic testing to identify low‐risk patients who may be exempt from chemotherapy requires a larger sample size for validation. Furthermore, the application of CDK4/6 inhibitors in adjuvant‐intensive endocrine therapy for MUMPC and prolongation of endocrine therapy currently lacks clear evidence and therefore requires further research.

Some studies have suggested that neoadjuvant chemotherapy or a combination of anti‐HER2 therapies can be employed for the treatment of triple‐negative PMC and HER‐2 overexpression PMC.^37^ None of the patients included in this study achieved a pathological complete response following neoadjuvant therapy, which was attributed to a limited reduction in the tumor volume of the mucous components. Further imaging studies are warranted to enhance the efficacy of neoadjuvant therapy for mucous cancer. Given the rarity of these cases, currently, there is no evidence of neoadjuvant therapy for MUMPC, and no studies have investigated its impact on OS and DFS.

Regarding the factors influencing the prognosis of MUMPC, to date, most studies have primarily focused on analyzing its pathological features, with limited reports on its prognostic implications. Micropapillary carcinoma is characterized by extensive lymphatic invasion, resulting in numerous lymph node metastases and early recurrence in the skin and chest wall.^38^ Therefore, it is crucial to identify micropapillary structures, as they may indicate heightened tumor aggressiveness and influence treatment decisions.

By analyzing the prognostic factors of MUMPC, Shet et al.^13^ identified the histological grade, lymph node metastasis, irregular tumor boundaries, and the presence or absence of micropapillary structures as significant factors influencing OS and DFS. A retrospective study conducted by Liu et al.^12^ demonstrated a significant decrease in the rates of OS and DFS among patients with MUMPC compared with those with PMC. Furthermore, the presence of micropapillary features was identified as an independent risk factor for DFS. Our findings indicate that the prognosis of patients with MUMPC is significantly influenced by the clinical TNM stage and lymph node metastasis (*p* < 0.001). Notably, lymph node metastasis was an independent prognostic risk factor in patients with MUMPC (*p* < 0.001). Furthermore, lymph node metastasis is the most pivotal clinicopathological feature of MUMPC and has a profound effect on patient outcomes. Consequently, individualized analyses should be conducted to guide the selection of surgical approaches and systemic treatment strategies for patients with lymph node metastasis.

In summary, it is crucial to investigate the morphological and biological characteristics of MUMPC thoroughly. In this retrospective study, we conducted a comparative analysis of the clinicopathological features of MUMPC and PMC, examined the prognostic factors influencing MUMPC, and enhanced our understanding of MUMPC. The lymph node stage was the most crucial clinicopathological feature of MUMPC. Furthermore, the endocrine treatment strategy is an independent risk factor affecting the prognosis of MUMPC. A more precise classification of MUMPC would facilitate hierarchical management and enhance the development of personalized treatment strategies. However, the genetic analysis of MUMPC requires subsequent genomic studies involving larger cohorts to reveal the morphological, clinical, and genetic characteristics associated with MUMPC.

## AUTHOR CONTRIBUTIONS

Conception and design: Beibei YANG, Menglu SHEN, Meng WANG, and Jing ZHAO. Administrative support: Beibei YANG, Meng WANG, and Jing ZHAO. Provision of study materials or patients: Beibei YANG, Menglu SHEN, Bo SUN, and Meng WANG. Data collection and assembly: Beibei YANG and Menglu SHEN. Data analysis and interpretation: All authors. Manuscript writing: All authors. Final approval of the manuscript: All authors.

## CONFLICT OF INTEREST STATEMENT

The authors declare that the research was conducted in the absence of any commercial or financial relationships that could be construed as potential conflicts of interest.

## Data Availability

The data that support the findings of this study are available from the corresponding author upon reasonable request.
